# Are power calculations useful? A multicentre neuroimaging study

**DOI:** 10.1002/hbm.22465

**Published:** 2014-02-19

**Authors:** John Suckling, Julian Henty, Christine Ecker, Sean C. Deoni, Michael V. Lombardo, Simon Baron‐Cohen, Peter Jezzard, Anna Barnes, Bhismadev Chakrabarti, Cinly Ooi, Meng‐Chuan Lai, Steven C. Williams, Declan G.M. Murphy, Edward Bullmore, for the MRC AIMS Consortium

**Affiliations:** ^1^ Brain Mapping Unit Department of Psychiatry University of Cambridge Cambridge United Kingdom; ^2^ Behavioural and Clinical Neuroscience Institute University of Cambridge Cambridge United Kingdom; ^3^ Cambridge and Peterborough Foundation NHS Trust Cambridge United Kingdom; ^4^ Sackler Institute for Translational Neurodevelopment and Department of Forensic and Neurodevelopmental Sciences Institute of Psychiatry, King's College London UK; ^5^ Division of Engineering Brown University Providence Rhode Island; ^6^ Autism Research Centre Department of Psychiatry University of Cambridge Cambridge United Kingdom; ^7^ Nuffield Department of Clinical Neurosciences University of Oxford Oxford United Kingdom; ^8^ Institute of Nuclear Medicine, University College London Hospitals London United Kingdom; ^9^ Centre for Integrative Neuroscience and Neurodynamics, School of Psychology and Clinical Language Sciences, University of Reading Reading United Kingdom; ^10^ Centre for Neuroimaging Sciences King's College London Institute of Psychiatry London United Kingdom; ^11^ Clinical Unit Cambridge, GlaxoSmithKline Ltd., Addenbrooke's Hospital Cambridge United Kingdom

**Keywords:** power calculations, neuroimaging, multicentre

## Abstract

There are now many reports of imaging experiments with small cohorts of typical participants that precede large‐scale, often multicentre studies of psychiatric and neurological disorders. Data from these calibration experiments are sufficient to make estimates of statistical power and predictions of sample size and minimum observable effect sizes. In this technical note, we suggest how previously reported voxel‐based power calculations can support decision making in the design, execution and analysis of cross‐sectional multicentre imaging studies. The choice of MRI acquisition sequence, distribution of recruitment across acquisition centres, and changes to the registration method applied during data analysis are considered as examples. The consequences of modification are explored in quantitative terms by assessing the impact on sample size for a fixed effect size and detectable effect size for a fixed sample size. The calibration experiment dataset used for illustration was a precursor to the now complete Medical Research Council Autism Imaging Multicentre Study (MRC‐AIMS). Validation of the voxel‐based power calculations is made by comparing the predicted values from the calibration experiment with those observed in MRC‐AIMS. The effect of non‐linear mappings during image registration to a standard stereotactic space on the prediction is explored with reference to the amount of local deformation. In summary, power calculations offer a validated, quantitative means of making informed choices on important factors that influence the outcome of studies that consume significant resources. *Hum Brain Mapp 35:3569–3577, 2014*. © **2014 The Authors. Human Brain Mapping Published by Wiley Periodicals, Inc.**

## INTRODUCTION

Power calculations are both applauded as key to proper study management through prior estimation of appropriate sample sizes [Lenth, 2001, 2007] and derided as fallacious when interpreting non‐significant results [Hoenig and Heisey, 2002]. However, they may offer a tangible, meaningful approach from which crucial decisions on study design, execution and analysis can be made. In particular, where the derivation of outcome variables for the primary statistical tests occurs in multiple steps, such as the processing of both structural and functional MRI data, power calculations also serve to quantitatively compare alternative configurations of processing pipelines.

We have previously described image‐based power calculations for multicentre neuroimaging studies with Type I errors controlled by the false discovery rate [Suckling et al., 2010]. Predictions of power, sample size and minimum observable effect size (difference in group means) are available across a range of common study designs. Within‐centre variances that include both the between‐subject and residual error variances at that centre, upon which power calculations are based, are estimated empirically from a calibration experiment. This experiment precedes the main study and involves MRI assessment at each centre of a cohort of typical controls with similar demographic characteristics as the target populations in the main study and in a manner which parallels its design and data acquisition protocols [Brown et al., 2011; Costafreda et al., 2007; Gountouna et al., 2010; Magnotta and Friedman, 2006; Shokouhi et al., 2011; Zou et al., 2005].

In this technical note, we describe data collection and analysis from a calibration experiment undertaken to support the Medical Research Council Autism Imaging Multicentre Study (MRC‐AIMS), a large‐sample cross‐sectional study of adult males with autism spectrum condition (ASC) conducted at three centres in the United Kingdom. The objective of MRC‐AIMS was to map differences in cognition and brain structure associated with ASC and their inter‐relationship. In support of this goal, the calibration experiment contributed to the conduct, analysis and interpretation of the main study by:

- Comparison of voxel‐based morphometry (VBM) analysis derived from two MRI acquisition sequences that depict structural anatomy of the brain at high resolution, and
- Observing the effects of recruitment profiles between‐centres on statistical power.

Results from the MRC‐AIMS structural MRI datasets have now been reported [Ecker et al., 2013, 2012]. It is thus possible to compare predictions of minimum observable effect sizes from the calibration experiment with the actual effect sizes obtained in the main study. In doing so we validate the power calculations previously reported [Suckling et al., 2010] and make some more general observations on the effect of non‐linear registration methods that are now recommended for VBM [Klein et al., 2009].

## MATERIALS AND METHODS

### Participants

#### Calibration study

Six participants (three males and three females) were scanned once at each centre with contemporary MRI machines operating at 3T and fitted with an eight‐channel receive‐only RT head coil: GE Medical Systems HDx, Department of Radiology, University of Cambridge (Centre 1); GE Medical Systems HDx, Centre for Neuroimaging Sciences, Institute of Psychiatry, Kings College London (Centre 2) and Siemens Medical Systems Tim Trio, FMRIB Centre, University of Oxford, Oxford (Centre 3).

#### MRC‐AIMS

Eighty‐nine male right‐handed adults with ASC (mean age 26 ± 7 years; range 18–43 years) and 89 matched typical controls (mean age 28 ± 6 years; range 18–43 years) were recruited and assessed at one of the three centres. Approximately equal ratios of cases to controls were recruited at each site: Cambridge: 30:32 (ASC: controls); London: 41:41 and Oxford: 18:16. ASC participants were diagnosed with autism according to ICD‐10 research criteria and then confirmed using the Autism Diagnostic Interview‐Revised [Lord et al., 1994].

Both studies were given ethical approval by the National Research Ethics Committee, Suffolk, UK. All volunteers in both the calibration experiment and MRC‐AIMS gave written informed consent.

### MRI Data Acquisition

Quantitative images of the spin‐lattice relaxation time, *T*_1_, were acquired using the driven equilibrium single‐pulse observation of *T*_1_ (DESPOT1) sequence [Deoni, 2007; Deoni et al., 2008]. This method derives an estimate of *T*_1_ from a series of spoiled gradient recalled (SPGR) and fully balanced steady‐state free precession images acquired over a range of flip angles. A common sagitally oriented field of view was used for the acquisitions. Centre‐specific sequence parameters are listed in Table 1. Acquisition time was 12 min and 23 s. Data were acquired from six participants at all centres; however, one dataset acquired from Oxford was discarded due to excessive head motion.

**Table 1:** Center specific parameters for the DESPOT1 sequence

Center	Field of view	Image matrix	TE (ms)	TR (ms)	FA (deg)	Bandwidth (Hz/pixel)
London	25 cm^2^ × 17 cm	256^2^ × 176	3.74	8.01	18,4	177
Cambridge	25 cm^2^ × 17 cm	256^2^ × 176	3.74	8.01	18,4	177
Oxford	25 cm^2^ × 16 cm	256^2^ × 160	4.80	9.10	20,4	400

TE = echo time; TR = repetition time; FA = flip angle.

Images acquired from each participant as part of the DESPOT1 acquisition protocol were co‐registered by affine transform to account for participant motion during the scanning session. Estimates of *T*_1_ at each voxel were then estimated [Deoni, 2007]. In brief, *T*_1_‐weighted inversion recovery images were simulated based on the pre‐computed *T*_1_ maps to optimise signal intensities, *S*, for accurate computational segmentation. At each voxel, the signal was calculated using the solution of the Bloch equation:

$$S=\rho \left(1-2e^{-\text{TI}\;/T_{1}}+e^{-\text{TR}\;/T_{1}}\right),$$

where TI = 850 ms, TR = 1,800 ms and the scaling constant *ρ* = 10,000. This combination of parameters results in good contrast between deep and cortical grey and white matter contrast. These simulated *T*_1_‐weighted images are not modulated by *B*_0_ and *B*_1_ field inhomogeneities, compensation having been introduced during estimation of *T*_1_. Thus, the subsequent segmentation did not require correction of field non‐uniformities [Sled et al., 1998].

At Centres 1 and 2 only, a *T*_1_‐weighted, high‐resolution three‐dimensional image was acquired with an Inversion Recovery SPGR (IRSPGR) sequence with the following parameters: voxel size = 1 mm × 1 mm × 1 mm; repetition time = 7.7 ms; echo time = 3.8 ms; inversion time = 450 ms; flip angle = 5°. Acquisition time was 2 min and 59 s.

### MRI Data Processing

All *T*_1_‐weighted images from DESPOT1 and IRSPGR sequences were processed with FSL v4.0 (http://www.fmrib.ox.ac.uk/fsl). Extracerebral tissues were removed with the Brain Extraction Tool [Smith, 2002], and maps of partial volume estimates of grey matter occupancy were calculated with FMRIB's Automated Segmentation Tool (FAST) [Zhang et al., 2001]. All grey matter images were initially linearly registered (FLIRT) [Jenkinson et al., 2002] and then non‐linearly registered (FNIRT) [Klein et al., 2009] to the stereotactic coordinate system of the Montreal Neurological Institute (MNI). Finally, to account for residual inter‐subject misregistration, the maps of partial volume estimates of grey matter were smoothed with a three‐dimensional Gaussian kernel with standard deviation = 4 mm (full width at half maximum = 9.4 mm).

### Power Calculations From the Calibration Study

Full details of the derivation of the voxel‐based power calculations are given in Suckling et al. [2010, 2008] and only an overview is presented here. Power is the probability of rejecting the null hypothesis when it is false. It is dependent upon the Type I error rate, effect size (difference in group means) and associated standard error. For a cross‐sectional study, (i.e., the two‐sample *t*‐test that was used to model the MRC‐AIMS) in which the differences in means of two groups (e.g., a patient and control groups) of equal size, *N*, are tested and with participants recruited at *C* centres with a proportion *Q_c_* at each centre:

$$Q_{c}=\frac{N_{c}}{\sum_{c=1}^{C}N_{c}}=\frac{N_{c}}{N},$$

where *N_c_* is the number of participants recruited in each group at each centre (i.e., assuming each centre recruits an equal number of participants from each group), and then the standard error is given by [Suckling et al., 2010]:

$${\text{SE}\;}^{2}={\left[\sum_{c=1}^{C}\frac{NQ_{c}}{2\sigma_{c}^{2}}\right]}^{-1},$$

where $\sigma_{c}^{2}$ is the within‐centre variance and includes both the between‐subject and residual error variances at that centre.

At each intra‐cerebral voxel in standard MNI space, the grey matter partial volume estimates were regressed onto a random‐effects model:

$$\begin{array}{c} y_{ic}=\mu_{0}+\mu_{i}+\beta_{c}+{ɛ}_{c}\\ {ɛ}_{c}\approx N\left(0,\sigma_{c}^{2}\right), \end{array}$$

where μ_i_ is the fixed effect for subject *i*, and β_*c*_ is the fixed effect for centre *c*. This model was fitted using the mixed model software lme [Pinheiro and Bates, 2000] in the R library of statistical software (http://www.r-project.org/).

From the model of power, the minimum observable effect size (difference in group means, *d*) was calculated at each intra‐cerebral voxel in standard MNI space after specifying, *C*, *Q_c_*, *N*, the acceptable level of Type I errors (α) and the acceptable level of Type II errors (β; fixed at 0.2 throughout this analysis). Similarly, specifying *d*, *C*, *Q_c_* and β, the minimum sample size per group was also calculated on a voxelwise basis.

Statistical thresholds on Type I errors (α) may be corrected in the power calculations for multiple comparisons using the false discovery rate correction [Suckling et al., 2010]. However, for this analysis, simple uncorrected statistical thresholds at α < 0.001 were used to indentify regions with large values of effect size for comparison of predicted and observed values. The largest effect sizes in the MRC‐AIMS were selected as the ability to make accurate predictions in these regions is clearly of greatest interest. As the size of a ROI increases, the regional mean effect sizes tend to the mean of the overall sample, which for a normally distributed data is zero. Similarly, selecting individual voxels where there is little or no effect yields a large proportion of regions with effect sizes near zero. On the contrary, predictions of minimum observed effect size based on estimates of within‐centre variance from the calibration study will always be >0. In such cases, the comparison between predicted and observed values of effect size would not be challenging to the technique.

Patterns of significant between‐group differences using appropriate corrections for multiple comparisons are reported elsewhere [Ecker et al., 2012].

## RESULTS

### Comparison of MRI Acquisition Sequences and Registration Techniques

**Figure 1:**
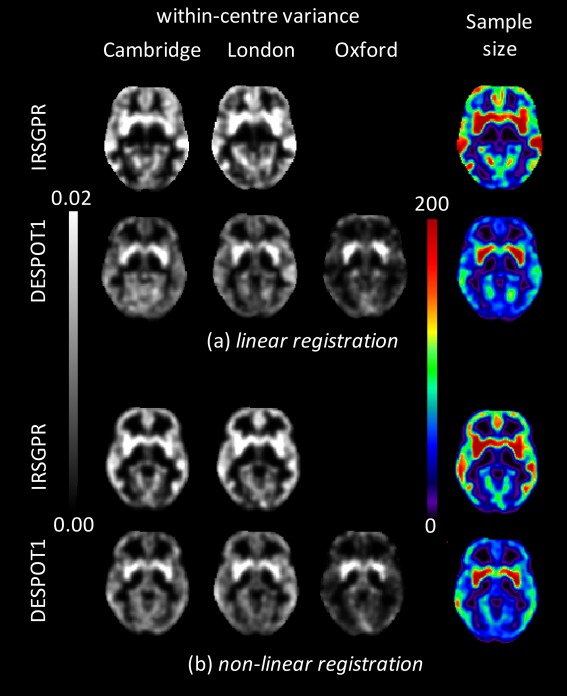
Within‐centre variances from each participating centre for DESPOT1 and IRSPGR sequences (where available) using (**a**) linear registration and (**b**) non‐linear registration of the individual images to standard stereotactic (MNI) space. Right‐hand column is the minimum sample size required to observe an effect size (difference in means) of *d* = 0.06.

Maps of within‐centre variance were generated for each centre, for each MRI acquisition and for each method of registration to standard MNI space (Fig. 1). Similar to previous results in an independently acquired calibration study, using grey matter segmentations of IRSPGR *T*_1_‐weighted sequence [Suckling et al., 2010], sub‐cortical structures display the greatest variance relative to areas of the neocortex, which is relatively spatially homogeneous. Segmentations from the DESPOT1 acquisitions also have elevated values of within‐centre variance in sub‐cortical structures, although the ratio relative to the neocortex is greater. This effect was particularly pronounced in data from Centre 3.

Two example regions in which grey matter differences associated with ASC have previously been reported [Rojas et al., 2006] were identified by anatomical atlas [Tzourio‐Mazoyer et al., 2002] as representative of regions where there is a large (bilateral putamen) and small (bilateral fusiform gyrus) difference in within‐centre variance between centres operating machines from different manufacturers. Regions of interest were created from the voxels within the atlas regions that had grey matter probabilities of >0.5. Ratios of mean within‐centre variance in bilateral putamen to that in fusiform gyrus calculated from segmentations of DESPOT1 acquisitions were 1.05, 1.35 and 3.05 following linear registration and 1.02, 1.16 and 2.70 following non‐linear registration for Centres 1, 2 and 3, respectively. By way of comparison, these ratios for the segmentations from the IRSPGR sequence were 0.99 and 0.88 following linear registration and 0.86 and 0.87 following non‐linear registration for Centres 1 and 2, respectively.

Power calculation estimates of minimum sample size for segmentations from both DESPOT1 and IRSPGR acquisitions were made using the following parameters: a nominal value of *d* = 0.06 [Suckling et al., 2010], *C* = 2 (only Centres 1 and 2 having acquired both sequences) and *Q_c_* = (0.5, 0.5) (i.e., equal distribution of participants across both centres). Example slices are shown in Figure 1. The minimum sample sizes estimated to observe this effect size with DESPOT1 segmentations were 101 and 124 following linear registration and 97 and 109 following non‐linear registration for regions of bilateral putamen and fusiform gyrus, respectively. Similarly, the minimum sample sizes for IRSPGR segmentations were 162 and 172 following linear registration and 146 and 126 following non‐linear registration for regions of bilateral putamen and fusiform gyrus, respectively.

Based on these results, although acknowledging that they only include two of three centres, the DESPOT1 sequence was selected as the primary outcome variable for MRC‐AIMS using non‐linear registration to map data into a standard stereotactic space.

### Influence of Recruitment Strategies Across Centres

Inspection of within‐centre variance (Fig. 1) makes clear the differences in the spatial distribution between Centres 1 and 2 that operated MRI scanners from the same manufacturer and Centre 3. Thus, for a systematic investigation of the consequences, the allocation of participants to the centres was varied.

**Figure 2:**
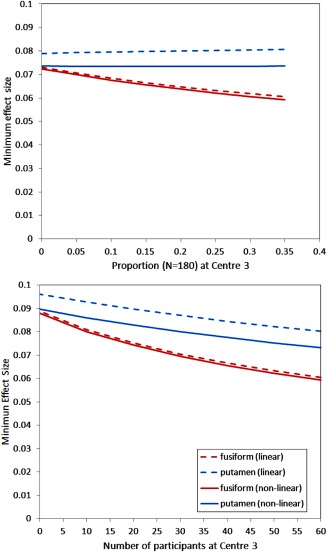
Minimum observable effect size estimated from segmentations of DESPOT1 acquisitions in sub‐cortical and cortical brain regions with images registered to standard MNI space by linear and non‐linear mappings as a function of (**a**) the proportion of the total sample of 180 participants attending centre 3 and (**b**) holding the number of participants attending centres 1 and 2 constant and varying the number of participants attending centre 3.

The sample size was fixed at 90 per group, and the proportion attending Centre 3 varied from *Q_c_* = 0.0 (no participants attend Centre 3) to *Q_c_* = 0.33 (equal number of participants attend all centres). The minimum effect sizes were then calculated with *C* = 3 for images registered with both a linear mapping only and with the addition of a non‐linear mapping (Fig. 2).

The minimum effect sizes in the putamen were largely unaffected by the distribution of recruitment across centres. However, a reduction (i.e., improvement) in minimum effect size was observed in the fusiform gyrus with increased proportion of attendance at Centre 3.

A second set of simulations estimated the minimum effect sizes after fixing the number of participants attending Centres 1 and 2 combined at 60 per group and then increasing the number of participants attending Centre 3 from 0 to 60 (i.e., from 120 to 180 participants in total).

Increasing the number of participants, unsurprisingly, reduces the minimum observable effect sizes. However, the difference between the minimum effect sizes in putamen and fusiform gyrus regions diverges as the number attending Centre 3 increases.

Across both simulations, minimum effect sizes were lower (i.e., improved) with non‐linear when compared with linear mappings, with the difference greater in the putamen.

### Validation of Power Calculation Predictions

The validity of the predictions made by the power calculations was tested once MRC‐AIMS was completed and reported. Predicted and observed effect sizes should have a monotonically increasing relationship, with predictions of minimum effect size less than the observed value at any intra‐cerebral location. Ideally, all points on a plot of observed against predicted values should lie above the line of identity.

To obtain the observed values of effect size from MRC‐AIMS, the F‐map corresponding to the between‐group analysis of grey matter segmentations from DESPOT1 acquisitions was thresholded at α < 0.001 uncorrected and aggregated into three‐dimensional clusters. This simple threshold was used for this experiment merely to generate a sufficiently large number of clusters for comparison with predicted minimum effect sizes from the calibration study. The resulting pattern will not be interpreted and thus the precise value of the threshold is unimportant. The observed effect sizes were then calculated from the absolute value of the difference in group means of grey matter within the cluster, averaged over the voxels that it contains.

To obtain the predicted effect sizes, a threshold of α < 0.001 was used in a power calculation simulating the MRC‐AIMS using the now known number and ratios of participants at each centre, creating a minimum effect size map. In those clusters identified from the statistical thresholding of the between‐group MRC‐AIMS F‐map (above), the predicted minimum effect sizes were calculated as the means from each cluster.

**Figure 3:**
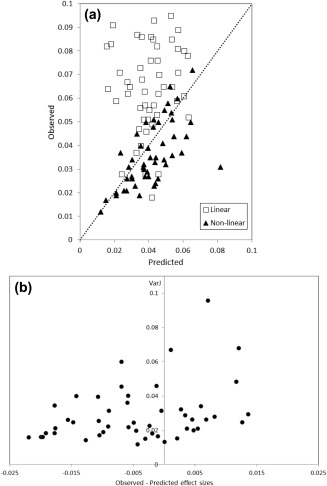
(**a**) Predicted effect sizes from power calculations against those observed from MRC‐AIMS in clusters identified by a statistical threshold of *α* < 0.001 uncorrected on a between‐group test of the MRC‐AIMS dataset. Results from both linear and non‐linear registration techniques are displayed, as is the line of identity (i.e., prediction = observation). (**b**) The variance of the Jacobian determinant following non‐linear registration against the observed–predicted effect sizes for each cluster. An outlying point at predicted–observed effect size = −0.052 is omitted from the figure.

Plots of the predicted against observed effect sizes are shown in Figure 3 for both linear and non‐linear registration techniques. Linear mapping produces observed effect sizes that almost exclusively (i.e., 48 of 51 regions) lie above that predicted, and thus power calculations in this instance are well validated. However, for non‐linear mapping, although there is high correlation between predicted and observed values (*R* = 0.684, *P* < 10^−6^), in 34 of 49 regions, the predicted values are overestimates of effect size. To explore why this might be the case, the clusterwise variance of the Jacobian determinant resulting from the non‐linear mapping was extracted and compared with the differences between the observed and predicted effect sizes for each cluster by a linear model. For a single cluster, the value of the observed–predicted effect sizes was >4.5 standard deviations from the mean. This cluster was also located on the edge of the parenchyma of the brain. It was thus considered an outlier. With this point omitted, the linear model was significant [*F*(1,47) = 4.53; *P* = 0.039; with the outlier included *F*(1,48) = 4.20; *P* = 0.046]. The relationship was positive, that is, the greater the observed value of the effect size exceeds that predicted, the greater is the local variance of warping (i.e., less smooth local features of the grey matter are associated with improved predictive performance), and vice versa. This test was non‐significant [*F*(1,47) = 2.70; *P* = 0.107; with the outlier included *F*(1,48) = 2.95; *P* = 0.093), when repeated with the clusterwise mean Jacobian determinant. Similar results derived independently from the regions of the anatomical atlas [Tzourio‐Mazoyer et al., 2002] are given in the Supporting Information.

## DISCUSSION

This article describes the application of previously reported power calculations for imaging studies [Suckling et al., 2010, 2012, 2008] to highlight how they may go beyond estimation of sample sizes to contribute to decision making on key design parameters of recruitment, data acquisition and data analysis. Decision support takes the form of comparisons of estimates of sample size and minimum effect size.

Selection of a MRI imaging sequence is a decision prior to opening the study that once made is largely inflexible and therefore of great importance. Newly developed sequences may offer significant advantages in terms of contrast, geometric distortion, signal homogeneity and so on. Often however, this may come at a price of additional scan time. In the example given here, DESPOT1 acquisition times relative to the alternative IRSPGR sequence is ≈4. This is offset by a reduction in sample size of 0.6–0.7 dependent on the location in the brain and the details of the registration. Thus, the total amount of acquisition time associated with the two sequences can be calculated and compared. Once the costs of recruiting and assessing additional participants were factored in, the evidence favoured the DESPOT1 sequence for the MRC‐AIMS. Of course, there are good technical reasons for choosing the DESPOT1 sequence, not least the reduced spatial inhomogeneity of *B*_1_, and such factors also need to be taken into account.

Estimates of within‐centre variance from participating centres permit the exploration of recruitment profiles during the main study, and specifically what effect they will have on recruitment rates. For the DESPOT1 sequence centre 3 was identified (Fig. 1) as having a profile of within‐centre variance that differed considerably from the other two centres, most likely accounted for by the different scanner manufacturer at that centre. However, altering the ratios of participants attending the centres suggested that there would only be a slight penalty in terms of an increase in the minimum observable effect size in sub‐cortical areas, which is more than offset by a reduction in cortical areas (Fig. 2). Increasing sample size improves overall performance in both cortical and sub‐cortical regions. It is interesting to note that the greatest improvement occurs with only a few participants assessed at Centre 3, after which the rate of improvement declines (Fig. 2). As the number of participants attending Centre 3 increases, it decreases as a proportion of the entire sample, and the effect of lower within‐centre variance at that centre diminishes.

Statistical power models make the assumption that the ratio of participants in each group is balanced at each centre, which is an important design feature for unbiased analysis. Furthermore, it makes no mention of changes in the direction of the effect at each centre (i.e., a qualitative centre × group interaction) [Suckling et al., 2012] that can profoundly alter interpretation of the overall result. Making suitable estimates of the effect size, *d*, generally poses difficulties. Neuroimaging studies of ASC have not reported the group mean volumes from regions of significant difference necessary to calculate the between‐group differences required for the power calculations, but rather *t*‐ or *Z*‐values which are obviously normalised by the associated standard error. Furthermore, the value of *d* is the smallest effect size observable, rather than the mean or some other measure of centrality, and is a global value across the entire brain parenchyma and thus does not reflect bounds on *d* that may occur due to the local anatomy. In summary, *d* cannot be estimated with reliable accuracy or generality, and thus in this study, a nominal value was used based on similar estimates made for other disorders [Suckling et al. 2010]. Having said that, the results presented here that impact on the design of the study are comparisons of sequences and profiles of recruitment across the centres. Variations in *d* would alter the specific values produced by the power calculations, but crucially do not alter the inferences made when comparing the relative values across sequences or profiles.

Once data collection is complete, the results from calibration studies can still be helpful in determining parameters of the data‐processing pipelines. Image registration is integral to neuroimaging as a precursor to voxel‐based statistical comparisons and the precise algorithm used in mapping from the acquisition space of the individual to the standard space of the group strongly influence outcomes [Klein et al., 2009; Suckling et al., 2006]. With power calculations it is possible to quantify the consequences of any changes in the pipelines and thus to better assess the relative benefits. The example given in this article compares the linear mapping (FLIRT) with a non‐linear mapping (FNIRT). Figure 2 demonstrates that a non‐linear mapping is not detrimental to performance but that improvements are dependent on brain location with very little difference between mappings seen in cortical areas, where the topology is relatively smooth, when compared with sub‐cortical areas that have greater changes in image contrast associated with grey/white matter boundaries.

Confirming the accuracy of power calculations gives credence to the technique and confidence in the values predicted for future studies. Data from MRC‐AIMS, now complete, are an opportunity to assess performance of the predictions by comparison of estimated and observed effect sizes from the independent datasets acquired in the calibration and main studies, respectively. With linear registration, only three of 51 clusters with large effects had observed effect sizes less than those predicted, substantiating the measurements from calibration studies and power calculations as a highly accurate technique. When non‐linear registration methods are used, the results are somewhat more equivocal. Although there is a highly significant linear relationship between predicted and observed effect sizes, 70% of clusters have predictions of a minimum value greater than those observed.

Why this might be the case was explored through analysis of the Jacobian determinant, which measures the amount of expansion or contraction a voxel undergoes during non‐linear mapping to, in this case, a standard stereotactic space. In general, clusters with greater variance in the Jacobian determinant were associated with positive differences between the observed and predicted values of effect size (i.e., observed > predicted). In other words, areas of the brain that contained more small‐scale features had more accurate registration of specific anatomical features at a given location resulting in better alignment across datasets. Conversely, smooth areas have more variable registration across datasets, and the attendant misalignment leads to poorer performance when comparing predicted with observed effect sizes. The nature of the local deformation—expansion or contraction—is not strongly coupled to this effect. The corollary of this proposition is that linear registration is overall very stable across datasets, although the accuracy of registration at any location is unlikely to be as good as when a locally deformable mapping is undertaken.

In this technical note, the performance was tested of image‐based power calculations, reported previously, that draw upon data acquired in a calibration experiment that precedes a main study. The participants used in the calibration experiment should be similar in demographic profile as those recruited to any subsequent study which it supports, although it is important to ensure that the two samples are independent to avoid inflation of Type I errors in the main study [Brown et al., 2009; Miller, 2005]. The accuracy of the predictions of power calculations has been demonstrated here to be excellent, perhaps surprisingly so given the number of assumptions involved in power calculations. This is testament to the maturity of MRI instrumentation available at the participating centres, as well as more widely to the neuroimaging community. On the basis of this finding, we recommend the use of these techniques as a way of quantifying the effects of parameters in the design of the study, as well as effects in post‐processing.

## Supporting information

Supplementary Information Figure 1A.

Supplementary Information Figure 1B.
