# Supplementary figures and images for: Are power calculations useful? A multicentre neuroimaging study
Source: Hum Brain Mapp. 2014 Feb 19;35(8):3569–77. doi: 10.1002/hbm.22465 (PMC4282319; doi:10.1002/hbm.22465)

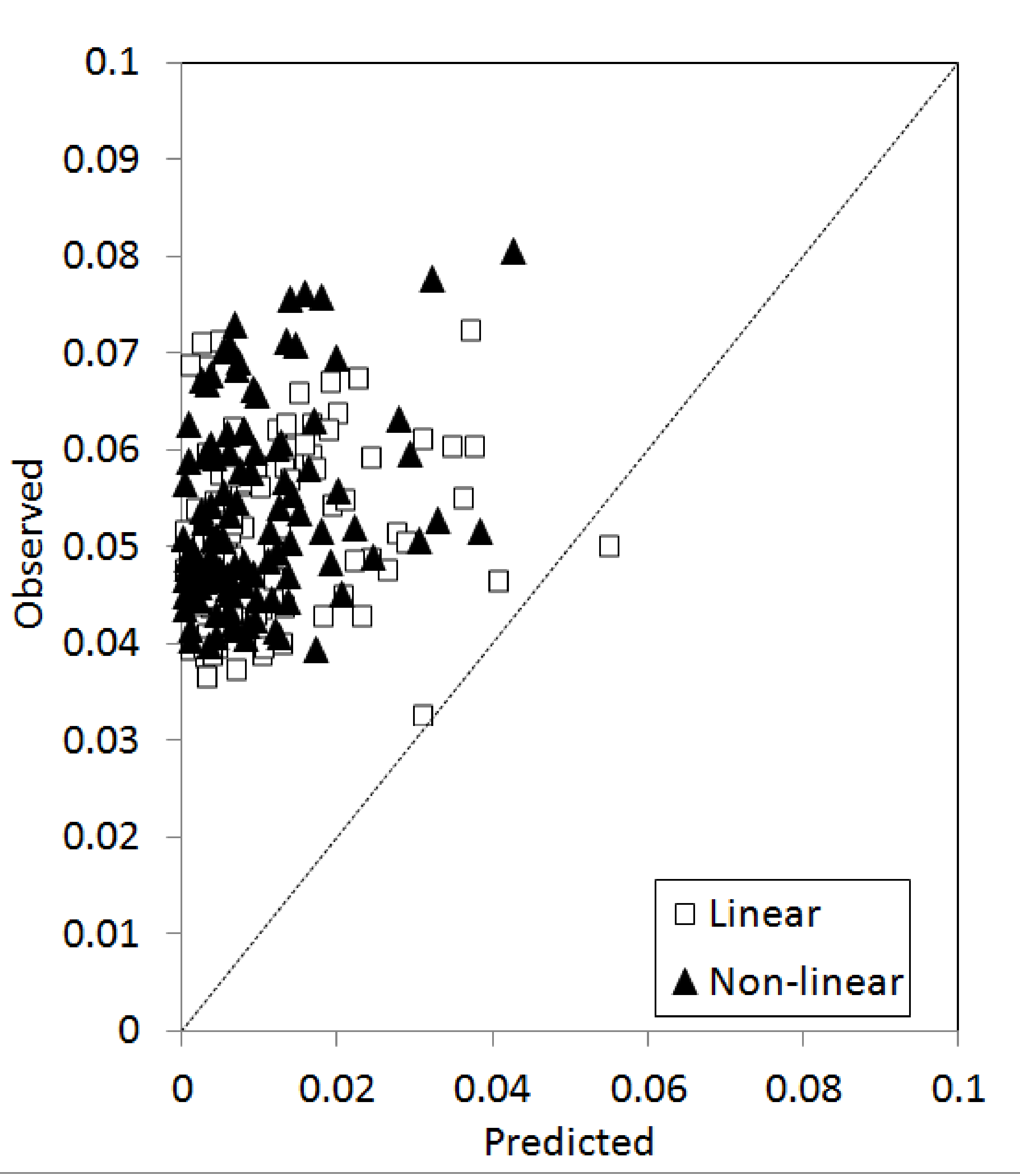

Supplement: Supplementary file 1 — Supplementary Information Figure 1A. [file HBM-35-3569-s001.tif]

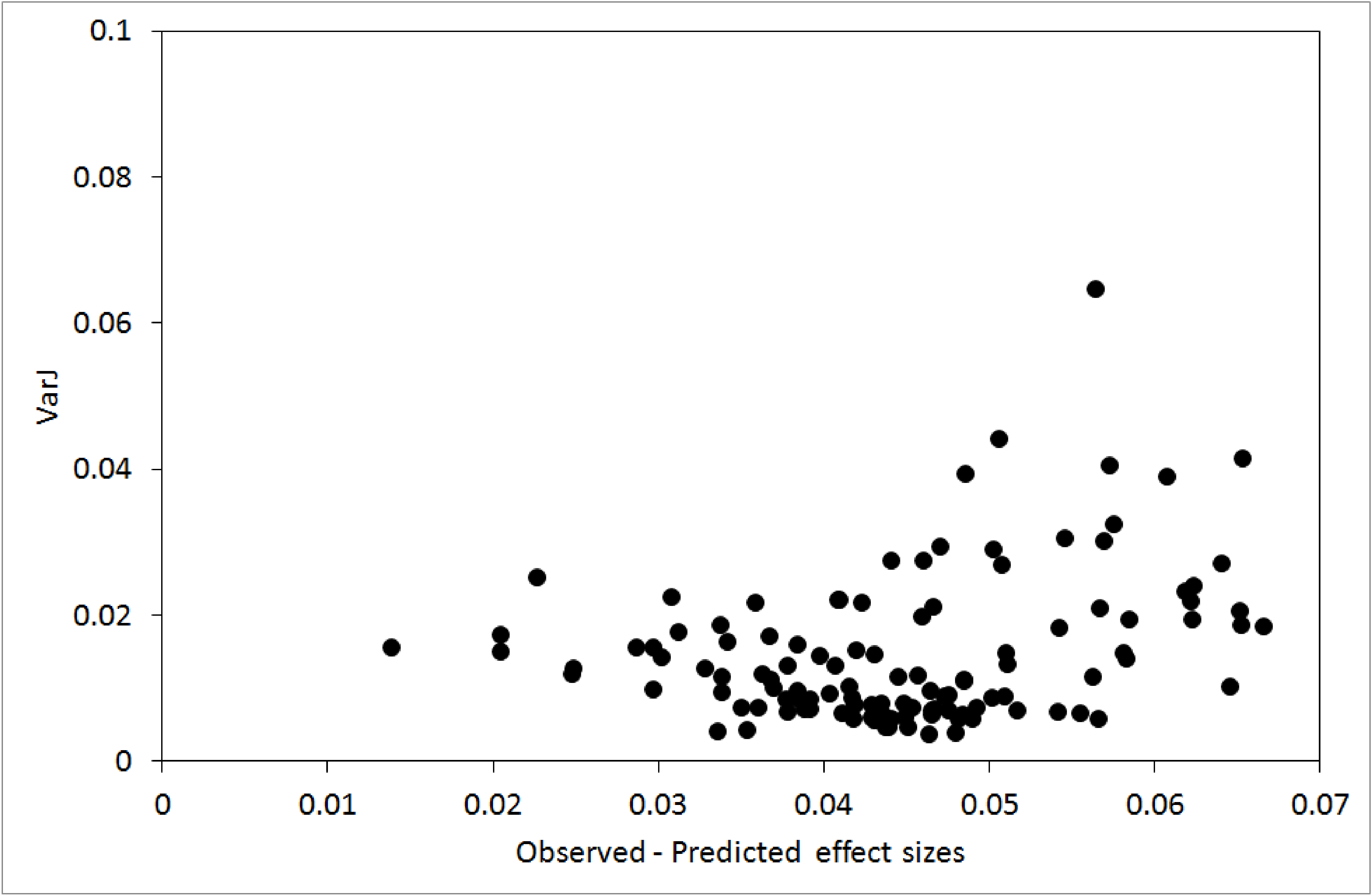

Supplement: Supplementary file 2 — Supplementary Information Figure 1B. [file HBM-35-3569-s002.tif]
